# Quality of *Pinus* sp. pellets with kraft lignin and starch addition

**DOI:** 10.1038/s41598-020-78918-7

**Published:** 2021-01-13

**Authors:** Paula Gabriella Surdi de Castro, Humberto Fauller de Siqueira, Vinícius Resende de Castro, Antônio José Vinha Zanuncio, José Cola Zanuncio, Matheus da Silva Berger, Francisco Damião Rodrigues Martins, Angélica de Cássia Oliveira Carneiro, Jorge Gominho, Solange de Oliveira Araújo

**Affiliations:** 1 Departamento de Engenharia Florestal, Universidade Federal de Viçosa, Viçosa, 36570-900 Brazil; 2 Instituto de Ciências Agrárias, Universidade Federal de Uberlândia, Monte Carmelo, 38500-000 Brazil; 3 Departamento de Entomologia/BIOAGRO, Universidade Federal de Viçosa, Viçosa, 36570-900 Brazil; 4 Centro de Estudos Florestais, Instituto Superior de Agronomia, Universidade de Lisboa, Lisboa, 1349-017 Portugal

**Keywords:** Renewable energy, Forestry

## Abstract

Pellets are widely used for power generation because they use renewable raw material with easy storage, transport and high energy density. However, the structural fragility, disintegrating during handling, transport and storage, is one of the main problems of pellets, but the addition of binders/additives can minimize this fragility. The objective of this study was to evaluate the properties of wood pellets with the addition of starch (corn and wheat) and kraft lignin in different proportions. Pellets were produced with the addition of starch (wheat and corn) and kraft lignin in the proportions of 1, 2, 3, 4 and 5% in relation to the mass of wood particles of *Pinus* sp., with 12% moisture (dry basis), classified in 3 and 1 mm sieves and compacted in a pelleting press in the laboratory, according to European standard EN 14961-2. Physical and mechanical properties of the pellets were evaluated and their densitometric profiles obtained from the Faxitron LX-60 X-ray equipment. Corn starch and kraft lignin additives at 4% improved pellet properties (density, fines and hardness), reducing their losses during handling, storage and transport.

## Introduction

Fuel pellets are widely used in Europe as an alternative to firewood, due to their ease of storage and high energy density^1–3^. The fragility of the pellet structure facilitates its disintegration, generating fines (crushed or powdered material) during handling and transport, which is the main problem of this material^4–6^. Organic additives such as starch from corn, manioc, pea starch, potato, sweet potato, rice, wheat, or yam as well as kraft lignin, may reduce pellet cracking and disintegration^7–9^.

The starch applied with water vapor should be added for the melting and gelatinization of the particles during pellet production^7^. The biomass heating, with a moisture content of 8–12% in the dry basis, alters the pellet particles, denaturing the proteins and increasing their hardness and quality^10–12^. In addition to its binding action, starch lubricates the pelletizing matrix and facilitates the flow of the densified raw material during pellet production^6^.

Kraft lignin, a residue from the wood pulping process, may increase the pellet’s mechanical strength and energy properties^13–15^. This material has potential because it is a waste product from the pulp production and can be obtained at affordable prices. Due to its chemical structure, lignin has found a number of applications. It can be used in cement composite fabrication^16^; as a filler for polymers^17,18^; in bio-based composite thin sheet films^19^; it was evaluated as an active material in II generation glucose biosensor^20^ and as component of adhesives^21,22^.

The quality of the raw material affects that of the pellets^23^, and the bonds between its particles must be smooth to reduce fissures, cracking or disintegration during handling, transport and storage^6^. The pellet surface should appear, to the naked eye, to be solid and with well-bonded particles without micro-cracks^24^. A weak cohesion between its particles reduces the mechanical strength and increases pellet cracking^2^. X-ray micro tomography and X-ray densitometry can analyze the internal structure of the material, enabling a more detailed analysis of different agroforestry products such as pellets and briquettes^25^.

The wood product quality can be evaluated using non-destructive methods such as the digital X-ray images used in fields such as dentistry, orthopedics, zoology and zootechnology^26^. X-ray densitometry has been used to characterize and evaluate the deterioration of the eucalyptus wood due to white rot fungi, and to detect the limits of the heartwood-sapwood, the effect of forest management on the wood properties, the annual production of biomass and the relation with its anatomical structure^27–29^. However, this technique is under-used for evaluating the internal density of solid biofuels such as briquettes and pellets^25,30,31^.

The objective of this study was to use the gravimetric method and X-ray densitometry to determine the apparent density and density profiles of *Pinus* sp. pellets with the addition of starch (corn and wheat) and kraft lignin in different proportions, as well as to measure their fines and hardness.

- We intend, therefore, to answer the following questions:
- How do additions of starch and kraft lignin to pellets affect apparent density?
- How do effects of starch and kraft lignin supply on pellets properties like fines content and hardness?
- How do the density apparent vary along the pellets longitudinal axis?

## Results and discussion

The fines content of the pellets, agglutinated with wheat starch and kraft lignin (both at 4%), was 125 higher and 75% lower than in the control, respectively (Table 1). The fines generation of the pellets in all treatments was lower than 1% (0.03 to 0.27%) and, therefore, they met the marketing standard EN 14961-2^32^.

**Table 1:** Fine content (%), hardness (%), bulk density (g m^−3^), apparent density (g m^−3^) by gravimetric method and apparent density (g m^−3^) by X-ray densitometry of *Pinus* wood pellets produced with different percentages of the additives (A) corn and wheat and kraft lignin and in the control.

Parameter	A%	Lignin	Corn	Wheat	Control
Fine content (%)	1	0.11 ± 0.08aA	0.16 ± 0.13aA	0.11 ± 0.04aA	0.12 ± 0.04
	2	0.10 ± 0.04aA	0.14 ± 0.02aA	0.12 ± 0.14aA	
	3	0.05 ± 0.03aA	0.13 ± 0.04aA	0.17 ± 0.10aA	
	4	0.03 ± 0.02bA	0.22 ± 0.11aA	0.27 ± 0.03aA	
	5	0.12 ± 0.06aA	0.05 ± 0.03aA	0.15 ± 0.03aA	
Hardness (%)	1	32.75 ± 4.72aA*	39.80 ± 4.37bA*	31.15 ± 3.44aA*	49.10 ± 9.75
	2	42.90 ± 6.79bB*	41.20 ± 3.02bA*	32.75 ± 7.82aA*	
	3	42.75 ± 4.90bB*	44.60 ± 4.95bA	28.50 ± 5.35aA*	
	4	45.45 ± 8.37bB	41.80 ± 6.20bA*	31.75 ± 4.18aA*	
	5	60.00 ± 8.49cC*	44.75 ± 6.09aA	39.95 ± 4.25bB*	
Bulk density (g.m^-3^)	1	0.64 ± 0.03bC*	0.66 ± 0.04aA	0.62 ± 0.01cA*	0.67 ± 0. 01
	2	0.64 ± 0.03aC*	0.64 ± 0.03aB*	0.60 ± 0.03bB*	
	3	0.69 ± 0.01aA*	0.61 ± 0.03bC*	0.61 ± 0.07bAB*	
	4	0.64 ± 0.05aC*	0.64 ± 0.03aB*	0.61 ± 0.09bAB*	
	5	0.67 ± 0.03aB	0.63 ± 0.01bB*	0.62 ± 0.04cA*	
Apparent density by grav. (g m^−3^)	1	1.20 ± 0.05aA*	1.21 ± 0.04aA*	1.17 ± 0.06aA	1.11 ± 0.11
	2	1.16 ± 0.07aA	1.22 ± 0.05aA*	1.16 ± 0.07aA	
	3	1.23 ± 0.03aA*	1.16 ± 0.07aA	1.15 ± 0.06bB	
	4	1.21 ± 0.01aA*	1.16 ± 0.06aA	1.18 ± 0.05aA	
	5	1.22 ± 0.05aA*	1.18 ± 0.06aA	1.17 ± 0.04aA	
Apparent density by X-ray dens. (g m^−3^)	1	1.26 ± 0.03bAB	1.31 ± 0.03bB	1.00 ± 0.05aA*	1.26 ± 0.04
	2	1.25 ± 0.05abAB	1.27 ± 0.03bB	1.22 ± 0.06aC	
	3	1.27 ± 0.04bAB	1.16 ± 0.03aA*	1.16 ± 0.03aB *	
	4	1.23 ± 0.03aA	1.29 ± 0.05bB	1.19 ± 0.06aBC*	
	5	1.28 ± 0.04bB	1.28 ± 0.04bB	1.18 ± 0.06aBC*	

Means followed by the same uppercase letter, per column, or lower case, per line, do not differ by Tukey test (*p* > 0.05). *Differences between treatments and the control by the Dunnett test (α = 0.05).

The lower values of the fines content of the pellets produced with kraft lignin are possibly due to the densification process of the pellet matrix with higher contents of this additive, generating pellets with better bonding characteristics between the particles and, consequently, less fines. In addition, lignin has a cementing action between the cells^9^ during the pressing process, and high temperature causes this compound to reach the glass transition stage, ensuring a strong bond between the particles^8,33^. Pellets with lower fines production during handling and transport should be preferred commercially^34^. The fines content increases with the moisture level of the material, causing cracks to exhaust gases, mainly water vapor, and, consequently, reducing their mechanical resistance during handling^35^. On the other hand, the low moisture content makes biomass compaction difficult, due to the water’s characteristic of helping the heat transfer and promoting lignin plasticization as a natural biomass binder^36^. The moisture content between 8 and 12% in the dry basis is ideal for reducing fines generation to within the European standard EN 14961-2^32^.

The hardness of the pellets was similar with the different percentages of corn starch, but it was higher with wheat starch (Table 1). The hardness increased by 22% when the percentage of kraft lignin reached 5%, in relation to the control. The hardness of the pellets with 3 and 5% of corn starch and 4% of kraft lignin was similar to the control.

The similar hardness of the pellets with the different percentages of wheat starch confirms studies that binders can reduce the mechanical properties of pellets at a higher moisture content, because water takes the place of hydrogen bonds, affecting cohesion between the particles^37^. Higher hardness affects pellet length, because the higher the hardness, the greater the breaking strength after contact with the pelletizing press knife^15^. In addition, pellets with lower hardness have points for water ingress, increasing the moisture content and consequently the breaking point and causing higher fine generation^38^. The higher hardness of pellets produced with 5% kraft lignin is possibly due to the decrease of their hygroscopic equilibrium moisture, due to the hydrophobic character of this compound. The kraft lignin residue is a compound of C–C and C–O–C phenylpropane units with low water relationship^39^. In addition, the constant pressing temperature of 120 °C plasticizes kraft lignin as an adhesive, increasing particle contact and reducing expansion due to lower hygroscopicity, consequently increasing hardness^40^. Kraft lignin, as an additive, facilitates the use of this residue and confers better properties to pellets by increasing their mechanical strength^13–15^.

The bulk density of pellets with 1% corn or wheat starches and 3% kraft lignin was higher than other mixtures (Table 1). The bulk density of kraft lignin pellets was higher than those with corn or wheat starch. The bulk density of pellets with 1% corn starch and 5% kraft lignin was lower than those with 3% lignin, which were denser than those with only wood (control).

The higher bulk density values for 3% kraft lignin pellets may be associated with a higher amount of lignin in the mixture (wood + additive), which plasticizes more efficiently, generating a smooth and uniform texture in the pellets and improving their density. The pelletizing matrix temperature influences the durability and bulk density of pellets^36^, as lignin is a natural wood binder and requires temperatures above the glass transition (75–100 °C) to produce bonding between the particles. Temperatures above 90 °C improve pellet characteristics, and require lower compaction pressure at increasing compaction matrix temperatures^4,41^. The lower density values of wheat starch pellets may be due to the high moisture content of the steam generated during the high temperatures in the compaction process (120 °C), causing micro-cracks in the pellet structure and reducing its density^35^. Starch acts as a lubricating agent in the pelletizing process, facilitating the flow of raw material through the pelletizing matrix^36^. The bulk density of the pellets was greater than the minimum required by the European Marketing Standard EN 14961-2^32^, equal to or greater than 0.60 g cm^−3^ in all treatments. This highlights the potential use of additives in pelletizing, which should be at most 2% relative to the dry mass of primary raw material.

The apparent density of pellets varied in a fashion similar to that of bulk density (Table 1), with no effect from the type and amount of additive added to the particles mass, comparing the three different additives and considering the same proportion used, except for pellets produced with 3% wheat starch, with lower apparent density. The apparent density of pellets produced with 1 and 2% corn starch and 1, 3, 4 and 5% kraft lignin was higher, and the other treatments were similar to the control (Table 1). Lignin and corn starch promoted better connection between particles, favoring biomass compaction and increasing pellet density.

The variation in the apparent density of the pellets, similar to that of bulk density between 1.15 g m^−3^ (3% wheat) and 1.23 g m^−3^ (3% lignin), is possibly due to the wheat starch gelatinization process starting at lower temperatures (± 70 °C) than that of corn starch (± 85 °C)^42^. This leads to the starch adhering to the pellet feeder system wall, reducing the proportion of additive that reaches the pelletizing matrix and consequently diminishing the unit density of the pellet. The higher apparent density of pellets produced with 1 and 2% corn starch and 1, 3, 4 and 5% kraft lignin is due to the lower rate of return of the pelletizing process and the higher molecular weight of the additives, influencing the pellet density^7,36^. Bulk density and apparent density determine pellet storage and transport conditions, and are directly related to energy density in those with 1 and 2% corn starch and 1, 3, 4 and 5% lignin, with higher density and a higher amount of energy per volume unit^43^.

The apparent density of the pellets produced with additives and evaluated by X-ray densitometry ranged from 1.00 to 1.31 g m^−3^ in their longitudinal axis (Table 1), with the lowest value for pellets produced with 1% wheat starch, and the highest value with 1% corn starch.

The lower apparent density values of wheat starch pellets can be associated with the presence of cracks (empty spaces), directly related to the susceptibility to rupture^2^. Low density peaks indicate small cracks that are attributed to a moisture content of the mixture or particle sizes inadequate for pelletizing^4^, affecting the physical properties of biomass densification^44^. The average apparent density of pellets is within the range established by the German standard DIN 51731, from 1.00 to 1.40 g m^−3^^45^.

**Figure 1:**
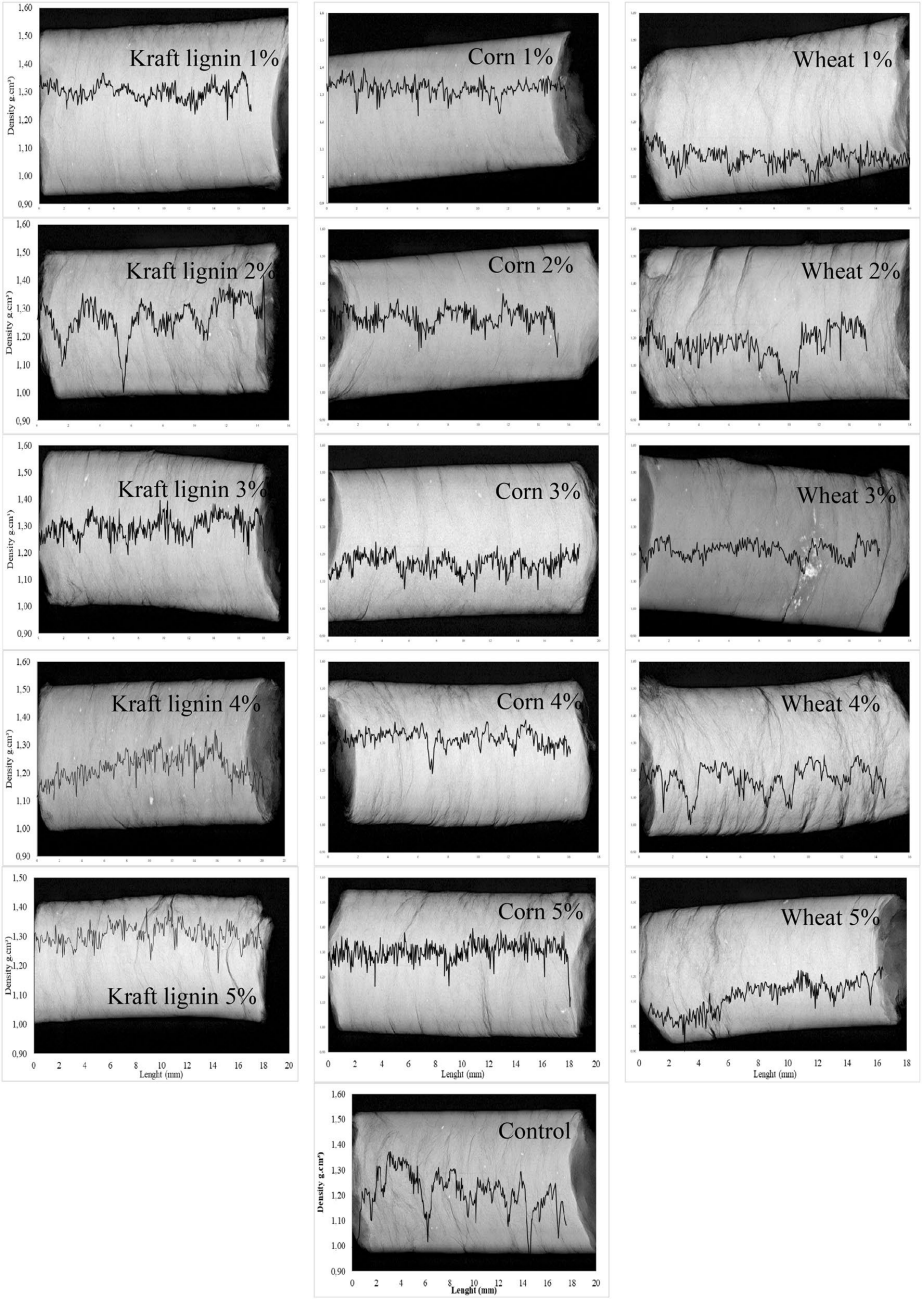
Longitudinal variation of pellet density with different proportions of the additives kraft lignin and corn and wheat starch.

Pellet density varied in longitudinal density profiles, with one uniform and one irregular pattern (Fig. 1). The apparent density variation of pellets produced without additives along the longitudinal axis (coefficient of variation of 5.29%) was higher. On the other hand, the apparent density variation of the profile (coefficient of variation of 4.19%) with additives was lower, showing greater cohesion between the particles and the additives. X-ray densitometry showed pellet density variations for all additives and in the control.

Uniform or irregular density patterns according to longitudinal pellet density profiles are due to variations in pellet internal density, which can be attributed to factors such as additive molecular weight, particle size, and temperature and pressure during pelletization^46–48^. Cracks are common in compacted material during pelletizing^4,6^, and can be attributed to inadequate pellet moisture content or particle sizes. The density of biomass varies with the moisture content^44^ and with the temperature strengthening the adhesion between the particles. Density profiles can explain the performance of pellets, whose cracks and high density variability affect their durability and final quality, since reductions in density are associated with cracks and, consequently, pellet breakage or rupture points, which can generate fines^5^. The apparent density of the pellets by gravimetric and X-ray densitometry, similar between treatments with additives, confirm that this technique, commonly used to evaluate the apparent density of materials and easier to apply than other methodologies, can be used to evaluate the quality of the pellets. Variations in the apparent density and longitudinal density profile obtained with the gravimetric and X-ray densitometry demonstrate that factors such as moisture, binder type, pressure and particle size interfere with the pelletizing process, causing variations in the material’s internal structure^46,47^. In addition, this technique accesses different parts of the pellet and therefore identifies point variations in the product density as reported for the 2% wheat starch pellet.

In conclusion, the additives reduced the fines content and increased the hardness and density of the pellets. Therefore, they have the potential to produce pellets with greater resistance to the transport, storage and handling processes. Apparent density along the longitudinal axis of the pellets without starch was higher. The apparent density of pellets containing starch increased the cohesion between the particles and reduced the density variation as shown by their densitometric profiles.

## Methods

### Pellet production

The experiment was installed in the municipality of Viçosa, Minas Gerais state, Brazil (20°45′14″S and 42°52′54″W).

**Figure 2:**
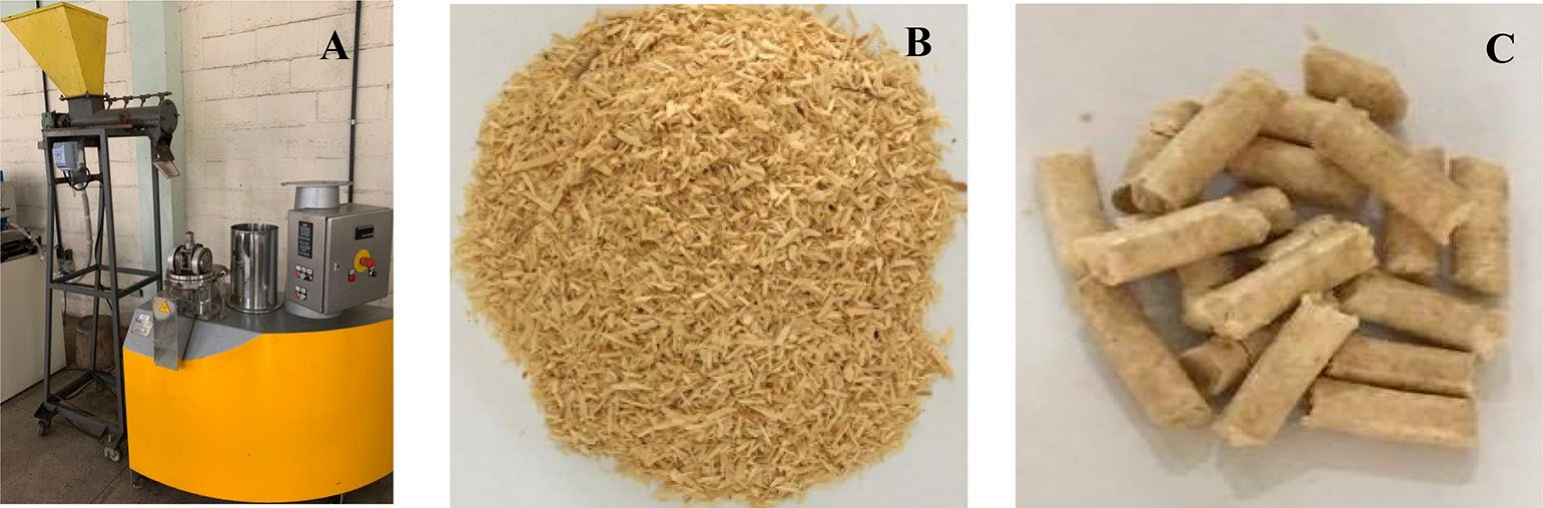
Pellets production. (**A**) Pelletizing machine press by Amandus Kahl, model 14–175. (**B**) Particles of *Pinus* sp. (**C**) Pellets produced.

Sawdust residues from headrig log breakdown of *Pinus* sp. were air-dried to a moisture content of approximately 20% (dry basis). The particles of these residues were classified using overlapping sieves, with the fraction that went through the 3.0 mm sieve and was then retained in the 1.0 mm sieve being used in the experiment (Fig. 2B).

The pellets were produced in a laboratory pelletizing machine press manufactured by Amandus Kahl, model 14–175 (Germany), with a production capacity of 30 kg h^−1^ (Fig. 2A). The average pelletizing temperature was 100 ± 5 °C. The pelletizing matrix was pre-heated in oil at 200 °C for approximately 30 min.

The pelletizing machine had a system with a motor reducer, a speed controller and a continuous screw. Four nozzles installed along the continuous screw injected water vapor produced by autoclaving at a pressure of 0.8 kgf/cm^2^.

Pellets were produced with the addition of corn and wheat starch and kraft lignin in the proportions of 1, 2, 3, 4 and 5% each in relation to the *Pinus* sp. dry mass (Fig. 2C).

### Pellet properties

The fines content (particles smaller than 3.15 mm) was determined using the Holmen Ligno-Tester (United Kingdom), in accordance with EN 15210-1^34^.

The hardness of the pellets was determined in a diametric compression test in a manual hardness tester with a scale of 0–100 kgf, manufactured by Amandus Kahl (Germany). One pellet at a time was inserted into the hardness tester, receiving an increasing load until the sample was fractured. The maximum load was defined as what a pellet could withstand before breaking.

The pellet bulk density was obtained according to EN 15103^49^, with the samples conditioned at 65% relative humidity and 20 °C temperature.

The apparent relative density was obtained by immersing the pellet in mercury^50^.

### X-ray densitometry

**Figure 3:**
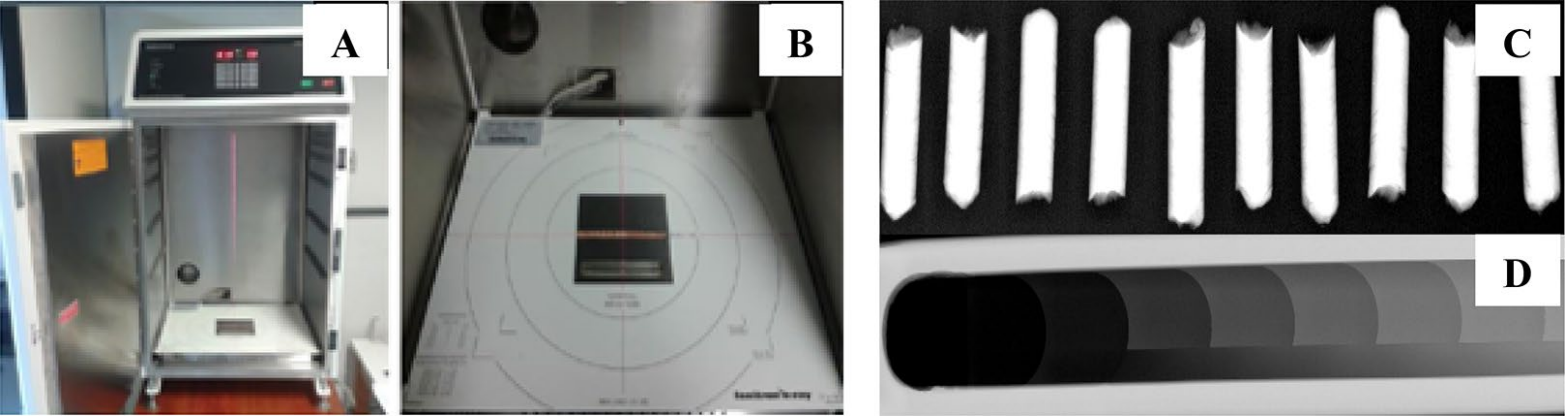
X-ray densitometry. (**A**) Faxitron model LX-60 cabinet X-ray system. (**B**) Shielded X-ray compartment. (**C**) Pellets. (**D**) Cellulose acetate calibration wedge.

The apparent density profile of the pellets was obtained in a Faxitron model LX-60 (United States of America) cabinet X-ray system (Fig. 3A). The pellets (Fig. 3C) and a cellulose acetate calibration wedge (Fig. 3D) were inserted into the shielded X-ray compartment (Fig. 3B), followed by calibration and an automatic reading (30 kV, 19 s), generating high-contrast and high-resolution digital images in the monitor screen. These images were saved in TIF format and analyzed using ImageJ software by transforming the gray scale of the wedge into apparent density values every 50 μm of distance along the longitudinal direction of the sample.

Ten pellets with 19 mm of length per treatment were analyzed and conditioned in an acclimatized chamber at 20 °C and 65% relative humidity, with 12–15% equilibrium moisture content, for 24 h.

### Statistical analysis

The apparent density, bulk density, fines content and hardness data were analyzed in a completely randomized design in a factorial arrangement with three additives (corn starch, wheat starch or kraft lignin) in five percentages (1, 2, 3, 4 and 5%), besides the control (pellets of pure *Pinus* sp. without additives). The averages were grouped using the Tukey test (*p* ≤ 0.05). The results considered the values of the control and were submitted to the Dunnett test (*p* ≤ 0.05).
